# A human right to assisted dying? Autonomy, dignity, and exceptions to the right to life

**DOI:** 10.1177/09697330251328655

**Published:** 2025-04-08

**Authors:** Jon Wittrock

**Affiliations:** 5264 Malmö University

**Keywords:** Four principles approach, Rights theory, Dignity in care, End of life issues, Palliative care, Autonomy

## Abstract

Debates on assisted dying remain controversial and call out for conceptual clarification. What is the moral basis for assessing competing arguments, and what is the best way to frame these arguments in terms of actual and potential human rights? This article aims to investigate whether autonomy alone suffices as a moral source for human rights and whether, on this basis, there should be a positive human right to assisted dying, and a negative human right to assist others in dying. Drawing upon discussions in political theory, medical ethics, and human rights scholarship, the article develops an account of autonomy as multidimensional and subject to trade-offs. Autonomy is divided into the dimensions of liberty, opportunity, capacity, and authenticity. Furthermore, there is a common intuition that human beings ought to be endowed with a domain of core autonomy that must never be compromised in any trade-off. This analytical framework is used to map conflicts and trade-offs concerning assisted dying. By way of conclusion, it is argued that autonomy suffices to describe what human rights protect, but not why they do so. Furthermore, it is argued that the terminology of rights used in debates on assisted dying risks misrepresenting what the debate is actually about, and that the debate should be framed in terms of the right to health and exceptions to the right to life, rather than general rights related to assisted dying. Thus, assisted dying should be seen as an extreme option, where death is not the end, but the means, and ought to be considered alongside other means, as a last resort, already in the legislative process.

## Introduction

Assisted dying continues to be a provocative and intensely debated topic, against the background of legal cases like that of Dániel András Karsai,^1^ Zoraya ter Beek,^2^ or recent criticisms of Canadian practices.^3^

*The right to die* is a term stemming from discussions about the cessation of life support from the 1970s to the 1990s. This was initially only a negative right to refuse medical interventions, following from principles of bodily integrity and autonomy.^4^ Subsequently, however, some have argued for a broader interpretation of a right to die, mirroring the right to life: as we are allowed to decide, within reasonable parameters, how to live, so, it has been argued, we should have the right to decide how to die.^5^

The term *assisted dying* refers both to *physician-assisted dying* and *voluntary active euthanasia*.^6^ In the former case, someone will intentionally end another’s life; in the latter case, a physician will provide the means for another to end their life. In both cases, this should be done as a consequence of a voluntary and competent request by the person whose life will end as a result of the administration of the means (normally drugs) in question.^6^ Assisted dying thus differs from *involuntary* (against someone’s will) and *non-voluntary* (lacking explicit consent) euthanasia.^6^ Common reasons for wanting assisted dying include severe pain and suffering, loss of physical and mental capacities, poor quality of life, and a desire for self-determination, including over one’s death.^6,7^ However, assisted dying need not necessarily be carried out by healthcare professionals, and we may conceive of a wide range of different reasons to want to die, from terminal illness to sexual desire or even mere curiosity. Do these reasons make a moral difference, and if so, why? Do some or all of them merit human rights protection?

In order to address these questions from a philosophical perspective, we first have to consider the ethical basis for human rights. In so doing, we may work more or less top-down or bottom-up, that is, either start out from a set premise and deduce rights, or try to induce from existing rights what they actually do, in a philosophical sense.^8^ I will focus on the latter approach, but in so doing, we may also reason about to what an extent existing and potential rights cohere with the dominant normative framework we uncover. Thus, in the following, drawing upon debates within political theory, human rights scholarship and medical ethics, I will present an analytical framework of *multidimensional autonomy* as that which human rights protect, and apply this framework to the topic of assisted dying. According to this interpretation, autonomy is neither a question of either/or, nor a question of maximizing some aspect of agency at the expense of all others. Rather, trade-offs might be called for not only between dimensions of autonomy in the present, but also between autonomy now, and autonomy later.

In the following, I thus aim to answer the following questions: *Is autonomy alone sufficient to provide a moral source for human rights? Should there be a negative right to assist others in dying, and/or a positive right to assisted dying?* By way of conclusion, I will argue that the dimension of *opportunity* (what actors may choose between) provides a key to unlock these debates, and briefly comment on the practical and legal implications of this analysis.

## Two levels of dignity

Rhetorically, human rights seem to be based on dignity, which is a concept that recurs in numerous key documents. The meaning of dignity, however, is not exactly clear, which has caused a considerable degree of confusion, not the least within medical ethics.^9,10^

Mann responds to the lack of conceptual clarity concerning dignity by both tentatively carrying out, and encouraging, empirical research into its meaning. Focusing on the experiences of violations of dignity, Mann proposes a taxonomy including, for example, physicians failing to make eye contact with patients or shake their hands; people being reduced, often but not necessarily pejoratively, to members of a group; a slap to the face; rape; being singled out (e.g., a child being told to stand in the corner of the room); or mistakenly standing out (e.g., someone applauding at the wrong moment in a concert) from a group or social norm. Violations of dignity, Mann speculates, may have negative health impacts.^11^ Building on Mann’s approach, Jacobson proposes an extensive list of dignity violations as well as strategies for the promotion of dignity; in the former case, ranging from rudeness to abjection, and in the latter, from contribution to love.^12^ Mann’s as well as Jacobson’s categories of dignity violations incorporate elements of coercion that clearly violate human rights and personal autonomy, but also components that point more towards a lack of recognition, and that are heavily reliant on context and interpretation, as well as elements that are simply irrelevant to serious ethical concerns (e.g., feeling undignified for applauding at the wrong moment).

It is thus hardly surprising that the concept of dignity, despite its rhetorical prominence, has drawn a lot of philosophical ire. Macklin, famously dismissing dignity as a “useless concept,” argues that the term is of little help when carrying out ethical analysis of medical activities, since it is either “hopelessly vague” or refers to “nothing more than a capacity for rational thought and action,” which is already “conveyed in the principle of respect for autonomy.”^13^ Perhaps autonomy could do the job dignity is supposed to do, but better?^14^ Respect for autonomy is indeed widely regarded as a crucial principle of medical ethics.^15,16^

When considering the relations between autonomy and dignity in ethical and human rights discourses, however, we might indicate quite different (and not mutually exclusive) things. We might argue that dignity, in this context, *actually means* autonomy. This need not imply that the *term dignity* should therefore be removed from key documents and replaced by the *term autonomy*; it may merely indicate that an account of autonomy tells us at least something, and perhaps everything, about what dignity *actually refers to*.

There are, however, further complications to consider, since dignity seems to play two very different roles here. *On the one hand*, dignity may be thought of as *that which human rights actually protect*. *On the other hand*, however, we may argue that dignity refers, not merely to *what* human rights protect, but to *why they protect it*—to the “moral ‘source’” of human rights.^17^ Recognizing this crucial point, several authors distinguish conceptually between different levels of dignity and introduce specific terms to refer to the kind of dignity which is supposed to provide a normative source for human rights protection, for example, *basic*, *human*, or *intrinsic* dignity.^18,11,19^ This crucial distinction can be visualized as follows (Table 1):

**Table 1.** Two levels of dignity.

Level 1 dignity	*What* human rights actually protect; often interpreted as *autonomy*
Level 2 dignity	The moral source which explains *why* we should have human rights

Confusingly, dignity as well as autonomy may thus mean different things, and play different roles, at different levels of argumentation. Notably, if level 2 dignity transcends autonomy, it may constrain it. For example, if human dignity is interpreted as deriving from the divine act of creation, we might well argue that God prohibits assisted dying, as being incompatible with the dignity He has bestowed upon us.^20^ This, however, can hardly be the dignity that is the source of human rights, since human rights are supposedly universal, and cannot credibly be based on Christian faith and theology alone.

In a survey of arguments used in the wake of Macklin’s attack on dignity, Hofmann notes that whereas many critics point to aspects of dignity extending beyond autonomy, or claim that autonomy, too, is vague, others argue that the very vagueness of dignity can be a strategic advantage, rendering it useful indeed in allowing for agreement between actors with different normative and ideological agendas.^21,22^ I propose to call this the notion of (level 2) dignity as a *strategic concept* which has allowed powerful people to agree on a rhetorically palatable but vague normative source for human rights: “we agree about the rights but on condition no one asks us why.”^23^ Thus level 2 dignity is violated whenever there is relevant agreement (e.g., between states or judges) that this is so; it is not violated if such an agreement is lacking (e.g., in the European system of human rights, if a question is deemed to fall within the margin of appreciation). This means that level 2 dignity is up for philosophical contestation, and depending on how it is defined (in terms of autonomy, utility, theology, etc.) we might reach different conclusions concerning level 1 dignity, and which rights people should and should not have. We might also take different stances on *how* an agreement on human rights and their justification *ought* to be reached, for example, through *overlapping* and *unforced* consensus.^24,25^

As for level 1 dignity, I agree that it is best understood as autonomy, but only within a framework of autonomy that is sufficiently complex not to obscure the difficult prioritizations and trade-offs involved in concrete decisions. In the following, I will apply this framework to the topic of assisted dying.

## Dimensions of autonomy and assisted dying

Either/or theories of autonomy, as Schwartz observes, tend to err in one of two ways: overly demanding theories of autonomy tend to gloss over the actual autonomy of real people, whereas excessively minimalistic definitions overlook the potential for autonomy to develop.^26^ Narrow normative theories of liberty, for their part, tend to propose accounts that fail to reach a desirable minimum of actual autonomy, for actual human beings.

In my understanding, autonomy is gradual and multidimensional, and its various constituent dimensions frequently contradict each other, calling for difficult trade-offs. It is obvious that we are not fully autonomous if we are constantly coerced; thus, if dignity is interpreted in terms of autonomy, we may think of human rights as protecting what Berlin calls *negative liberty*,^27^ In the following, I will simply use the term *liberty* as referring to the absence of threat and coercion by others. That this is a key aspect of debates on assisted dying probably goes without saying: we certainly do not want people to be euthanized against their will; however, coercion is relevant in prohibitions on assisted dying as well. Thus, a *negative right to assist* might imply that nobody should be prevented from directly or indirectly assisting another’s request for assistance in dying.^28^ This could of course be motivated by recourse to individual autonomy and a rejection of paternalism.

As for *positive* liberty, however, “being one’s own master”^29^ there are arguably several dimensions of presuppositions involved, for such a state of affairs to be truly desirable, for actual human beings. First, we obviously need *abilities to act*, or what I propose to call the dimension of *capacity*. Being immobilized and slowly approaching death hardly constitutes a desirable state of affairs, even if nobody coerces or threatens us to do anything. In my usage, a capacity refers to something we can do, if we are provided with the opportunity, in the external world, of doing so. Many capacities emerge over time, and can be lost, gradually or abruptly, and hopefully relearned. Pertaining to assisted dying, the capacity—psychologically and/or physically—of committing suicide is of obvious relevance. However, the gradual loss of capacities in general is often perceived as a major threat to human dignity. One study of residents in a nursing home found that “failing bodies were the most significant threat to their dignity, as loss of abilities was constantly progressing.”^30^ It is not surprising that a loss of capacities is one of the major reasons that people request assisted dying.^6,7,31^ To what an extent key, desirable capacities are universal and should be compiled into lists is a distinct question open to empirical investigations beyond the confines of this analysis. However, that governments ought to assist people in developing some key capacities is incorporated as a part of the human rights edifice, although the actual extent of what this implies is a subject of normative and political debate.^32^

Besides capacities, however, we also probably want access to a broad spectrum of *options to choose between*, or what I will call the dimension of *opportunity*. If there were nothing else in our world than ourselves and the resources and abilities we need to survive and perhaps to move around—we could envisage, for example, flying within a vast, dark space with a jetpack strapped to our back and an infinite source of nourishment—most people would agree that this would not be a situation in which we would be autonomous in any particularly desirable way. We want, not simply resources to consume—most obviously, gases to breathe, nutrition to eat, potable liquids to drink—but also entities and processes to perceive and interact with in a wider sense. Without these, there would be “nothing to do and nowhere to go, nothing to be and no-one to know.”^33^ Thus, even if we are able to do a lot, in the abstract, if we do not have access to a world of entities and experiences, our autonomy still arguably falls far short of being actually desirable. The range of our capacities impacts the range of our opportunities; for example, if we are unable to digest a certain substance, it does not constitute a desirable option for food or drink, for most of us. The opposite, however, is not true; our capacity for doing something does not automatically produce an opportunity to do it; the ability to digest food does not conjure up splendid dinners, and the ability to read does not print books. Nevertheless, the dimension of opportunity is frequently overlooked by analyses of liberty and autonomy, perhaps because we take it for granted that there simply is an external world full of opportunities. Nevertheless, without collective action, humanity as a whole may well lose access to valuable opportunities (cultural or natural), without which our actual autonomy would be diminished.

When it comes to debates on assisted dying, opportunities are of crucial importance: at the center of these debates emerge questions about which options are or should be available, to whom, and according to how stringent requirements. There are several arguments for a negative right to assist besides the mere fact that some are physically unable to commit suicide—for example, some may *approach* becoming unable to commit suicide, and may be forced to kill themselves earlier than they would have preferred, whereas others may resort to suicide in a way that is unpleasant to them and traumatic to witnesses.^5^ If there is a negative right to assist, should it be restricted to only pertain to those suffering from a terminal illness, or to anyone who simply decides that they want to die, for whatever reason? Should there be a *positive* right to assisted dying, implying publicly financed options? Should there perhaps be public as well as private alternatives? And what about the environment in which people die? In an article on the Dignitas house in Switzerland, a previous location is described as a “new flat in an industrial area” that “was so brutal in its simplicity that several relatives were horrified by the surroundings and one, Daniel Gall, was so upset that he wrote a book denouncing the experience…”^34^ Subsequently, the practice was moved to a more pleasing location. Furthermore, however, it has been convincingly argued that there is a danger of presenting assisted dying as merely one option, equivalent to others, and of neglecting the considerable resources already available besides assisted dying, within the confines of conventional practices.^4^ Some critics have, in the light of the problems of social deprivation observed amongst those who request assisted dying, pointed to the frankly terrifying risk “that death might serve as an alternative to provision of adequate social supports and other community-based programs.”^35^ More generally, there is the risk that the lack of crucial opportunities due to living conditions and socioeconomic status—for example, desirable options for housing, or access to quality healthcare options within reasonable time limits—might incline some, who might not otherwise have done so, towards seeking assisted dying.

Finally, then, there is the crucial question, acutely relevant to the topic of assisted dying, whether choices are genuine or not. Problems of ascertaining whether choices are genuine have, like so many other topics in medical ethics, been interpreted in terms of clashing definitions of dignity, when what is actually involved are, as far as I can see, different aspects of autonomy.^36^ Thus, decisions may be thought of as more or less genuine based on access to information as well as potential alternative courses of action (opportunity) as well as the absence of coercion (liberty) and the capacity for critical reflection on the short- and long-term consequences of different choices. What I propose to call the dimension of *authenticity* combines all of the preceding dimensions of autonomy when it comes to decision-making and is reminiscent of Griffin’s notion of normative agency as well as Raz’s conditions of autonomy.^37,38^ It should be noted, however, that I understand authenticity as a *threshold concept*: a decision may be considered *more or less* authentic, and when considering some standard, we are required to set criteria for what suffices to reach an acceptable threshold. Thus, Dworkin’s suggestion that autonomy could be grasped as the capacity for critical reflection upon one’s preferences would be framed, in my terms, as pointing towards a *possible* (and rather demanding) *threshold* for *authenticity*.^39^ Thus, my account of autonomy is neither *normal* nor *normative* but provides an analytical framework for *situating* different normative preferences or nonideal specifications.

Questions concerning authenticity have been particularly contested regarding instances of assisted dying of those who do *not* primarily suffer from problems of physical health. A key issue has been whether those suffering from personality disorders and psychiatric illness are affected in their very capacity for authentic decision-making, thus possibly meaning that their seemingly authentic—informed, consistent, uncoerced, and reflected—demands for assisted dying still fail to reach a desirable threshold for authenticity.^40^ For example, Ratcliffe argues that certain psychiatric conditions afflict those suffering from them with an inability to conceptualize positive change in their own capacities and opportunities as a realistic possibility.^41^

To conclude the above analysis, autonomy may be divided conceptually into four dimensions, liberty (freedom from threat and coercion), capacity (abilities to act if the opportunity arises), opportunity (the actual opportunities for choices available to an agent), and authenticity (the extent to which an agent’s choices are genuinely their own). These dimensions, and examples of questions pertaining to assisted dying, are summarized below, in Table 2:

**Table 2.** Dimensions of autonomy and assisted dying.

Liberty	Is there a prohibition on assisted dying, backed up by state coercion?
Capacity	Is someone able (physically, psychologically) to commit suicide?
Opportunity	Is assisted dying institutionally available and publicly financed?
Authenticity	Are requests for assisted dying genuine and based in informed reflection?

Autonomy conceived of in multidimensional terms is gradual: we can be *more or less* well informed, have more or less extensive *opportunities* to acquire information, and be more or less *capable* of assessing the implications of that information, and translate those implications into carefully reflected agency. We need to pay careful attention to the fact that autonomy is indeed not a matter of either/or; rather, it is more or less *spatially* and *temporally* extended, implying difficult trade-offs between dimensions, concerning the distribution of the presuppositions of autonomy between persons and nations, and between any configuration and distribution of autonomy in the present and its preservation over time. The theme of trade-offs will be further developed in the following section.

## Trade-offs and diffusion arguments

If we were to maximize some dimension of autonomy at the expense of all others, we would soon discover that the result would be a horrific existence. Maximum liberty would allow us to freely traverse an immense space of absolute darkness, and maximum capacity would make us gods without equals or hindrances; conversely, maximum opportunity would result in something akin to immurement, being overwhelmed and mobbed by other agents and entities. As for authenticity, it *has* to be a threshold concept, since maximizing authenticity, if this were even possible, would at best turn us into someone like *Funes the Memorious*, the figure imagined by Borges in one of his short stories, who remembers everything in extreme detail: we would be overburdened with information, and unable to make decisions.^42^ Rather than *maximum autonomy*, a desirable multidimensional autonomy implies boundaries and trade-offs concerning its constituent dimensions.

Tensions between dimensions of autonomy, however, are not mere matters of philosophical abstraction: taxation ultimately rests on coercion, which restricts liberty, but is arguably necessary for the provision of opportunities (e.g., access to healthcare) and assistance in the development of capacities (e.g., through education). Concerning the dimensions of autonomy outlined in the preceding analysis, there is a strong emphasis on liberty in the Universal Declaration on Human Rights (UDHR) and the International Covenant on Civil and Political Rights (ICCPR), with a greater emphasis on opportunity in the International Covenant on Economic, Social and Cultural Rights (ICESCR), although those divisions are not clear-cut, of course. There is generally less emphasis put on capacity, although some formulations are surprisingly far-reaching, perhaps most notably the right to education (UDHR, article 26).

There are also trade-offs to be made, however, between any configuration of autonomy *in the present*, and its preservation *over time*. Republican thinkers in particular have pointed to the crucial importance of the temporal aspect, that is, whence sources of coercion derive, and how individual and collective autonomy can be protected over time—although this is a matter of emphasis.^43^ There are thus spatial as well as temporal trade-offs pertaining potentially to any dimension of autonomy, as evidenced by debates on, for example, citizenship, conscription, and derogation. The International Bill of Human Rights enshrines the general principles of equality and universality in access to rights, but allows for an uneven distribution, spatially, and temporally, both due to emergencies and derogation in article 4 of the ICCPR, and in the context of the progressive realization of second generation rights, mentioned in article 2 of the ICESCR.

The problems of spatial and temporal trade-offs have often been handled using the terminology of dignity, even when the trade-off in question actually pertains to autonomy. For example, Beyleveld and Brownsword distinguish between human dignity as *empowerment* and *constraint*, respectively.^44^ The former lies in the background of the post-war development of human rights, whereas the latter, they argue, has risen to prominence more recently. Furthermore, they claim, the former is tied to individual autonomy, and thus a priority on informed consent, whereas the latter tends towards paternalism and related, socially imposed constraints on individual autonomy. They also develop their own interpretation of dignity as a virtue rooted in the recognition of and capacity to confront existential uncertainty. Applying this framework to the question of assisted dying, they conclude that dignity and autonomy are closely connected, and support assisted dying.^44^ However, as Beyeleveld and Brownsword refer to arguments allegedly venturing beyond issues of individual autonomy, and towards conceptions of dignity rooted in communitarian concerns*, these arguments, too, can be translated to arguments concerning individual autonomy*, as long as we interpret the latter as both multidimensional and subject to spatiotemporal trade-offs.

Many arguments invoking dignity as seemingly distinct from individual autonomy either take the form of, or could be translated to, concerns about the potential for a dangerous *diffusion*, that indeed comes to threaten individual autonomy *across space*, and *over time*. In other words, it is sometimes maintained that, if we allow for a certain practice that is itself consistent with, and indeed follows from, embracing individual autonomy, such a practice could, over time or in a different setting, give rise to a restriction of individual autonomy, calling for a temporal trade-off between autonomy now (of some individuals) and autonomy later (of other potential individuals). These types of arguments, notably, can be applied to self-regarding agency in two ways: firstly, we typically want to make sure that important decisions are not being made frivolously, but follow from a genuine preference that is stable over time; secondly, however, we might argue that even a self-regarding decision that meets our criteria of authenticity, could be risky in the sense of *other* people’s future autonomy. That there are legitimate trade-offs between autonomy now and autonomy later, and that autonomy right now might have to be restricted for the preservation of autonomy over time, is widely accepted. An “autonomy centric” approach need by no means be “atomistic.”^45^

Pertaining to assisted dying we may distinguish between *slippery slope* and *overspill* arguments, respectively. The first type worries that allowing for assisted dying within the limits of autonomy *now*, might result in involuntary or non-voluntary euthanasia *later*.^6^ The second type worries that if we allow for autonomous assisted dying, some people may be *pressured* into consenting to die, for the sake of others.^46^ These arguments are illustrated below, in Table 3:

**Table 3.** Diffusion arguments and assisted dying.

Slippery slope arguments	Even if it would be fully consistent with a desirable level of individual autonomy to allow assisted dying in the present…	…doing so might compromise a desirable level of individual autonomy in the future, since the criteria for euthanasia might expand in a dangerous direction, resulting in involuntary or non-voluntary euthanasia
Overspill arguments	Even if it would be fully consistent with a desirable level of individual autonomy to allow assisted dying in the present…	…doing so might result in some people consenting to die in order to please relatives, or because they come to see themselves as a burden on society

The topic of trade-offs gives rise to the question whether there is some sphere of autonomy that must always be maintained and never sacrificed, or what I propose to call *core autonomy*. This is not a question of one specific dimension of autonomy (e.g., liberty) but rather of a contested domain, open to interpretation, extending potentially across all dimensions. In the framework of human rights, there are, for example, the non-derogable rights listed in ICCPR 4:2 as well as the “minimum core obligations” mentioned in General Comment 3 of the Committee on Economic, Social and Cultural Rights. More broadly, concerns about privacy and bodily autonomy point to issues of core autonomy culturally, and in public and political debates. If we do have a sphere of core autonomy, however, that puts limits on what states are ever allowed to do, and puts in place positive obligations spelling out what they always have to do, this must plausibly be connected closely to some very important concerns. That matters of life and death belong to such concerns is probably uncontroversial, and if the question is one of assisted dying based in genuine consent, core autonomy appears to point in the direction of allowing, and perhaps providing, rather than prohibiting, assisted dying. It should be noted that we might hold that there is some kind of core *value* or *dignity* to life, that constrains what people may legitimately do even to themselves; but then we are not really referring to *autonomy*.

On the one hand, it is thus difficult to see how core autonomy would itself cancel out assisted dying, without drawing upon the kind of diffusion arguments presented above; although it does potentially add something to the discussion concerning *who* should carry out the assistance, implying that nobody should be forced to assist, rendering any positive right to assisted dying contingent on the willingness of others to assist.^5^ On the other hand, however, core autonomy, if taken simply in the sense of relating to something of great importance to people, does not in itself generate either negative or positive rights (e.g., I might wish to reside in my ancestral castle, but I have no absolute human right to do so). Furthermore, it is clear that human rights are supposed to address *important*, not trivial, concerns.^47^ They are not aiming to maximize our autonomy and provide us with the tools to do anything we might enjoy doing, even if it harms no one else, and even if it is tied to something of great importance to us.

## Conclusion

Whereas dignity functions as a rhetorical justification for human rights, I have argued that autonomy more plausibly describes *what* human rights protect, provided that we operate with a complex, multidimensional account of autonomy. Debates on assisted dying were then structured within this framework. It was observed that the *authenticity* of choices was a key aspect of debates on assisted dying, and that it in turn rested on the prerequisites of the absence of threat and coercion, as well as access to information and potential alternative courses of action, the capacity for critical reflection on short- and long-term consequences of different decisions, and the expression of a consistent preference over time. This implies that healthcare providers should seek to assess that requests for assisted dying are genuine, in terms of demonstrating a sufficient degree of these characteristics. Ultimately, however, it is up to the legislators to provide people with, or conversely withholding, desirable options and legitimate courses of action, and they should approach debates on assisted dying with a clear understanding on what is actually being considered.

We are now in a position to answer the questions stated at the outset. *Is autonomy alone sufficient to provide a moral source for human rights?* In human rights discourse, dignity jumps between all of the conceptual elements elucidated in the preceding analysis: it refers alternately or simultaneously to core autonomy, dimensions of autonomy, the extension of autonomy across space and over time, and the opaque underlying source justifying protection of all dimensions and rights. Given this multitude of meanings, it is little wonder that both sides in a conflict can claim, with good reasons, to defend human dignity. The preceding analysis, however, has strengthened the notion that autonomy does suffice to describe *what* human rights protect. At the very least, autonomy, if explicated as multidimensional and subject to spatial as well as temporal trade-offs, does suffice to cover key aspects of debates on assisted dying. Even when level 1 dignity is invoked as something allegedly venturing beyond the confines of individual autonomy, it can be translated back to claims concerning autonomy, as long as the latter is interpreted with sufficient complexity. This indicates that dignity can indeed be replaced with autonomy, as *that* which human rights protect. Doing so allows us, as I have attempted to demonstrate, to more clearly articulate the trade-offs involved in concrete decisions, and to outline and motivate prioritizations being made. Thus, it cannot be credibly maintained that autonomy, as I have interpreted it, is as vague as dignity. Level 2 dignity, however, remains obscure, to the extent that we try to interpret it as something more than agreement between relevant, powerful actors. There is thus no compelling reason to interpret it as *morally* constraining autonomy, although it will in fact constrain autonomy, if powerful actors agree that it should. This analysis is summarized below, in Table 4.

**Table 4.** The normative architecture of human rights.

Level 1 dignity: what human rights protect	Autonomy spatiotemporally extended in the dimensions of liberty, capacity, opportunity and authenticity, calling for trade-offs—between dimensions, and concerning any configuration of autonomy in the present, and its preservation over time—restricted by core autonomy (which must always be maintained and never violated)
Level 2 dignity: the moral source of human rights	A strategic concept that enables different actors to indicate agreement on an alleged moral source of human rights that remains constant regardless of the prioritizations and trade-offs involved in level 1 dignity

*Should there be a negative right to assist others in dying, and/or a positive right to assisted dying?* A positive right to assisted dying as well as a negative right to assist may appear to follow naturally from an insistence on the centrality of individual autonomy. It bears to be repeated, however, that human rights do not rely on preferences alone; there have to be convincingly strong *reasons* why certain preferences are *important*. The dilemma for defenders of a positive right to assisted dying, and of a negative right to assist, is this: if someone merely wishes to die in some specific way, or for some idiosyncratic reason, they have no convincing reasons either for requiring a positive right to assistance, or a negative right for another to assist them; if, however, they seek relief from suffering or loss of capacities, they *do* have strong reasons for requiring assistance, but then death is not the end but the means. The distinction is illustrated schematically in Table 5, below:

**Table 5.** Framing debates on assisted dying.

Primary rights focus	End	Means
Right to die	Death	Forms of assisted dying (and the criteria and processes involved)
Right to assisted dying		
Right to assist		
Right to health	Alleviating suffering and the loss of autonomy	A range of options in order of escalation towards assisted dying as an extreme option, possibly preceded, for example, by psychedelic drugs (and the processes and criteria involved)
Right to life (exceptions)		

Assisted dying, as discussed by scholars and medical professionals, concerns exceptions from the right to life, primarily aiming to alleviate pain, suffering, and the loss of autonomy. This implies that death should be viewed as a last resort, not merely of currently legal options, but already in the process of legislation (e.g., prohibiting all use of psychedelics in healthcare settings while legalizing assisted dying seems very strange; as if these were two disparate topics rather than potential, extreme options related to the same or similar health problems, and as if death were obviously the less risky option). Debating an actual, general right to die or assist another in dying might be philosophically interesting, but probably less compelling for non-philosophers.
